# The association between processes, structures and outcomes of secondary prevention care among VA ischemic heart disease patients

**DOI:** 10.1186/1471-2261-6-6

**Published:** 2006-02-09

**Authors:** P Michael Ho, Allan V Prochazka, David J Magid, Anne E Sales, Gary K Grunwald, Karl E Hammermeister, John S Rumsfeld

**Affiliations:** 1 Department of Medicine, University of Colorado Health Sciences Center, Denver, CO, USA; 2 Medical Service, Denver VA Medical Center, Denver, CO, USA; 3 Clinical Research Unit, Kaiser Permanente of Colorado, Denver, CO, USA; 4 Division of Emergency Medicine, University of Colorado Health Sciences Center, Denver, CO, USA; 5 Department of Biometrics and Preventive Medicine, University of Colorado Health Sciences Center, Denver, CO, USA; 6 Health Services Research and Development, VA Puget Sound Health Care System, Seattle, WA, USA; 7 Colorado Health Outcomes Program, University of Colorado Health Sciences Center, Denver, CO, USA

## Abstract

**Background:**

Hyperlipidemia and hypertension are well-established risk factors for recurrent cardiovascular events among patients with ischemic heart disease (IHD). Despite national recommendations, concordance with guidelines for LDL cholesterol and blood pressure remains inadequate. The objectives of this study were to 1) determine concordance rates with LDL cholesterol and BP recommendations; and 2) identify patient factors, processes and structures of care associated with guideline concordance among VA IHD patients.

**Methods:**

This was a cross sectional study of veterans with IHD from 8 VA hospitals. Outcomes were concordance with LDL guideline recommendations (LDL<100 mg/dl), and BP recommendations (<140/90 mm Hg). Cumulative logit and hierarchical logistic regression analyses were performed to identify patient factors, processes, and structures of care independently associated with guideline concordance.

**Results:**

Of 14,114 veterans with IHD, 55.7% had hypertension, 71.5% had hyperlipidemia, and 41.6% had both conditions. Guideline concordance for LDL and BP were 38.9% and 53.4%, respectively. However, only 21.9% of the patients achieved both LDL <100 mg/dl and BP <140/90 mm Hg. In multivariable analyses, patient factors including older age and the presence of vascular disease were associated with worse guideline concordance. In contrast, diabetes was associated with better guideline concordance. Several process of care variables, including higher number of outpatient visits, higher number of prescribed medications, and a recent cardiac hospitalization were associated with better guideline concordance. Among structures of care, having on-site cardiology was associated with a trend towards better guideline concordance.

**Conclusion:**

Guideline concordance with secondary prevention measures among IHD patients remains suboptimal. It is hoped that the findings of this study can serve as an impetus for quality improvement efforts to improve upon secondary prevention measures and reduce the morbidity and mortality of patients with known IHD.

## Background

Hyperlipidemia and hypertension are prevalent and often co-existing conditions among patients with ischemic heart disease (IHD) [1]. Prior studies have found significant gaps in the achievement of nationally recommended low-density lipoprotein (LDL) cholesterol and blood pressure (BP) levels [2-5]. A basic principle of prevention is that intensity of risk reduction therapy should be adjusted to a person's overall risk [6]. Within 6 years of an initial event, up to one-third of patients will have a recurrent myocardial infarction, and almost 50% of women will be disabled with congestive heart failure [7]. In addition, the risk of a future IHD event, according to the Framingham risk estimates, is based on multiple factors including blood pressure level, cholesterol level, smoking status, and age [8]. Therefore, given the high absolute risk of recurrent cardiovascular events among IHD patients, studies addressing secondary prevention care should encompass multiple risk factors (e.g., hyperlipidemia and hypertension). However, prior studies assessing guideline concordance with secondary prevention measures have focused mainly on either hyperlipidemia or hypertension alone within any one study.

To improve secondary prevention care, factors associated with guideline concordance or non-concordance need to be further elucidated. One approach is to use the processes, structures and outcomes of care model [9] developed by Donabedian. He linked the three elements by stating that "good structure increases the likelihood of good process, and good process increases the likelihood of a good outcome." This model can be used to identify specific factors that are associated with guideline non-concordance and these factors can serve as focus areas for quality improvement interventions. Accordingly, our objectives were to assess guideline concordance for secondary prevention care based on LDL cholesterol and blood pressure control and to identify specific patient, processes of care and structures of care factors associated with guideline concordance.

## Methods

### Study population

All active patients with IHD enrolled in primary care clinics in any one of eight Pacific Northwest Veterans Affairs (VA) hospital facilities in Veterans Integrated Service Network (VISN) 20 were included in the study. Patients with IHD were identified by any one or more of the following International Classification of Diseases-9th revision (ICD-9) diagnosis codes in the 24 months before the index date (October 1, 2000), based on a previously validated algorithm: 410.x, 411.x, 412, or 414.x [2,10]. We searched for these diagnosis codes in the active problem list file of the VA computerized medical record, the outpatient care (OPC) file, and the patient treatment file (PTF). The OPC file contains data on all outpatient visits, whereas the PTF file contains data on all inpatient encounters. These files are part of the Consumer Health Information & Performance Sets (CHIPS) Data Warehouse [11], which is a relational database mirroring the clinical information system residing at each VA Medical Center and outpatient facility in VISN 20.

Active patients were defined as being alive on October 1, 2000 (index date), and having at least one primary care clinic visit documented in the OPC file per year in each of the two previous years. A primary care visit was defined using the OPC file as any visit to one or more of the following clinics: General Internal Medicine, Women's Clinic, Primary Care/Medicine, or Geriatrics.

### Independent variables

Clinical characteristics were defined by ICD-9 codes within either the OPC and/or PTF files within the 15 months before the index date: percutaneous coronary intervention (PCI), coronary artery bypass graft (CABG) surgery, diabetes, hypertension, hyperlipidemia, chronic obstructive pulmonary disease, peripheral vascular disease, cerebrovascular disease, myocardial infarction, congestive heart failure, malignancy, smoking status, and renal disease. The distance to medical center was calculated using the longitude and latitude coordinates of a patient's home address, based on their zip code and the longitude and latitude coordinates [12] of the medical center based on the medical center zip code.

Recent cardiac hospitalization for an acute coronary syndrome (ACS) or coronary revascularization procedure(s) (i.e., PCI and/or CABG) were defined as occurring within the 15 months before the index date. Hospital admission for ACS was defined by a primary discharge diagnosis based on ICD-9 codes 410.x or 411.x in the PTF file. The number of outpatient clinic visits was aggregated over the 15 months before the index date.

Patients were defined as having a current medication prescription for the following classes of medications if they had a prescription written or renewed within the last 15 months before the index date: aspirin, beta-blockers, angiotensin-converting enzyme (ACE) inhibitors or angiotensin receptor blockers, calcium channel blockers, diuretics, and 3-hydroxy-3-methylglutaryl coenzyme-A reductase inhibitors (statins). Patients frequently obtain aspirin over-the-counter rather than at VA pharmacies because of the lower cost and therefore rates of aspirin use are likely to be higher compared to the pharmacy prescription data. The total number of medications was based on the sum of all active prescriptions for the patient within 6 months from the beginning of the study period.

Facility level data were obtained from the VISN Support Service Center website [13], a health care information and technical support organization serving the needs of the networks. Data on volume of primary care patients and visits were obtained for each of the eight facilities included in the analysis. Clinic volume was defined by the number of visits to the primary care clinics at each of the 8 hospitals in the study for the month of December 2000. The number of visits ranged from 3,821 to 18,613. On-site cardiologist was defined as the presence of a cardiologist based at a hospital. Five of the eight hospitals in the study had an on-site cardiologist.

### Dependent variables

The primary outcomes of interest were achievement of: 1) LDL <100 mg/dl; and 2) BP <140/90 mm Hg [6,14], consistent with national guidelines as treatment targets for patients with underlying IHD. The combined outcome was defined as achievement of both LDL <100 mg/dl and BP <140/90 mm Hg. Any lipid level measured within 15 months before the index date was included, and the most current LDL level was used in the analyses. A LDL measurement was available for 83.4% of the patients. The LDL outcome variable was categorized into 3-levels of guideline concordance with each lower category indicating better concordance compared to a higher category: 0) LDL measurement and LDL <100 mg/dl, 1) LDL measurement, but LDL≥100 mg/dl, and 2) no LDL measurement.

The majority of patients (94.9%) had at least one BP measurement within the six months prior to the index date. Among patients with two or more BP measurements, the BP readings were averaged to get a better reflection of the true blood pressure. The BP outcome variable was also categorized into 3-levels of guideline concordance with each lower category indicating better concordance compared to a higher category: 0) SBP<140 and DBP<90 mm Hg; 1) SBP≥140 or DBP≥90 mm Hg, and 2) no BP measurement.

### Statistical analysis

The main objectives of our study were to identify specific patient, processes of care and structures of care factors associated with guideline concordance. We hypothesized *a priori *that the following patient factors would be associated with worse guideline concordance: older age, longer distance from home to medical center, diabetes and vascular disease (i.e. peripheral and/or cerebrovascular disease). For processes of care, we hypothesized *a priori *that the following factors would be associated with worse guideline concordance: higher total number of medications, lower number of outpatient visits and recent hospitalization for a cardiac event. Hospitalizations and cardiac procedures were categorized as process variables because the primary outcomes of interest were achievement of LDL and BP goals. Finally, for structures of care, we hypothesized *a priori *that the following factors would be associated with worse guideline concordance: lower primary care clinic volume and facility does not have an on-site cardiologist.

In our multivariable regression analyses, the cumulative logit model was used because there were 3 categories in both outcome variables. This model reports the odds ratio, which estimates the probability that each subject is in a lower (i.e., better concordance) category compared to a higher (i.e., worse concordance) category of guideline concordance.

Since we were interested in the association between specific factors and guideline concordance, our multivariable risks models were constructed specifically to test the independent association between each of the hypothesis variables and guideline concordance, adjusting for baseline patient characteristics. To test the association between specific patient factors and guideline concordance, a baseline patient level risk model was constructed consisting of patient demographic factors, cardiac and non-cardiac co-morbidities as listed in Table 1. Backward selection was performed (p < 0.05 to stay in model) to identify a parsimonious group of patient level factors associated with guideline concordance for risk adjustment. Then, each candidate independent variable of interest for hypothesis testing was individually entered into this baseline risk model to determine its association with the outcome of interest. Odds ratio and 95% confidence intervals are reported for each of the significant variables in the risk models.

**Table 1:** Baseline characteristics of the study population (N = 14,114)

**Patient characteristics**	**N (%)**
Age, mean years (STD)^*#^	68.1 (10.5)
Male gender	13,802 (97.8%)
White race	12,040 (85.3%)
Married*	8,246 (58.4%)
Distance from home to medical center, mean miles (STD)	71.6 (203)
Prior myocardial infarction*	1,557 (11.0%)
Prior percutaneous coronary intervention^*#^	1,278 (9.0%)
Prior coronary artery bypass graft surgery^*#^	2,971 (21.0%)
Congestive heart failure^#^	2,954 (20.9%)
Diabetes*	4,939 (35.0%)
Hypertension^*#^	10,093 (71.5%)
Hyperlipidemia^*#^	7,856 (55.7%)
Obese (BMI>30 kg/m^2^)^#^	6,048 (42.8%)
Smoker	2,909 (20.6%)
Chronic obstructive pulmonary disease^#^	4,201 (29.8%)
Cerebrovascular disease^#^	1,542 (10.9%)
Peripheral vascular disease^#^	1,613 (11.4%)
Renal disease	845 (6.0%)
Malignancy^#^	1,711 (12.1%)

*Significantly associated with LDL guideline concordance

^#^Significantly associated with BP guideline concordance

Risk models for the process of care analysis were built in a similar fashion as described for patient factors. A baseline risk model consisting of all significant patient-level factors identified in the prior analyses and the primary facility where a patient received care was constructed for risk adjustment. Then each of the process of care hypothesis variables was individually entered into the risk model to test the independent association between the variable and the outcome of interest.

The structures of care analyses were performed at the facility level using a hierarchical logistic regression model with a random facility effect that accounts for the clustering of patients by facilities. Models were estimated using maximum likelihood with the SAS procedure NLMIXED [15]. Multivariable hierarchical regression models were built to test the hypothesis of interest, accounting for clustering of patients within sites.

Based on the multivariable analyses, we identified several patient factors and processes of care variables that were associated with guideline concordance. To enhance clinical interpretability of the results, we evaluated the association between the number of factors present versus guideline concordance, using the chi-square test to assess for a trend in this association.

All analyses were performed using SAS (Version 8.02, SAS Institute) [14]. The study was approved by the Colorado Multiple Institutional Review Board.

## Results

Overall, 14,114 patients were identified as having IHD (Table 1). The majority of the IHD patients were male and the mean age was 68.1 ± 10.5 (mean ± STD). These patients had a number of co-morbid conditions with 71.5% having a diagnosis of hypertension, 55.7% having a diagnosis of hyperlipidemia, and 41.6% having both of these conditions. They had frequent outpatient visits (mean 8.7 ± 7.0 visits) over a 15-month period and were prescribed multiple medications (mean of 11.2 ± 6.0 medications) (Table 2).

**Table 2:** Processes of care for the study population

**Processes of care**	**N (%)**
**Recent hospitalization**	
Acute coronary syndrome hospitalization	534 (3.8%)
Percutaneous coronary intervention	213 (1.5%)
Coronary artery bypass graft surgery	284 (2.0%)
**Outpatient visit history**	
Total number of visits, mean (STD)	8.7 (7.0)
Total number of primary care visits, mean (STD)	7.3 (6.2)
Total number of specialty care visits, mean (STD)	1.6 (3.3)
Primary care clinic visit	13,425 (95.1%)
Cardiology clinic visit	3,862 (27.4%)
**Medication history**	
Total number of medications, mean (STD)	11.2 (6.0)
Aspirin prescription	7,051 (50.0%)
ACEi or ARB prescription	8,024 (56.8%)
β-blocker prescription	8,209 (58.2%)
CCB prescription	5,238 (37.1%)
Diuretic prescription	6,650 (47.1%)
Any lipid-lowering agent prescription	9,020 (63.9%)
Statin prescription	8,060 (57.1%)

**Figure 1:**
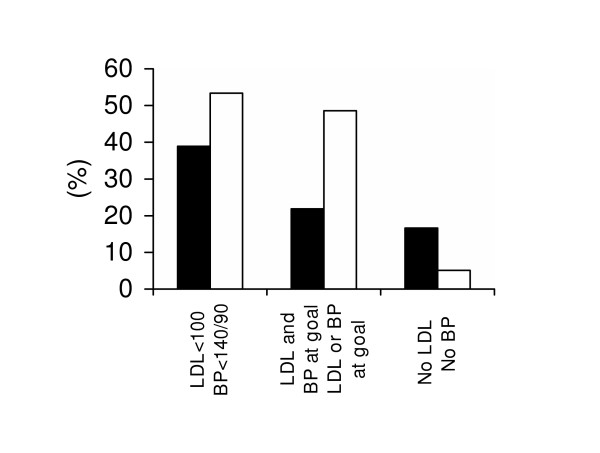
Guideline concordance for LDL cholesterol and blood pressure. * Patients were grouped into exclusive categories for "LDL and BP at goal' and 'LDL or BP at goal'. Therefore, patients at both LDL and BP goal were not placed in the numerator for the category 'LDL or BP at goal'.

Guideline concordance for LDL cholesterol and BP were suboptimal (Figure 1). Only 38.9% of the patients had a LDL<100 mg/dl and 16.6% of the patients did not have a current LDL measurement. For BP control, 53.4% of the patients had a BP<140/90 mm Hg and 5.1% of the patients did not have a current BP measurement. When guideline concordance was based on both achievement of LDL<100 mg/dl and BP<140/90 mm Hg, only 21.9% of the patients met these targets. A significant proportion of patients, 29.5%, met neither LDL nor BP goals.

The multivariable findings are presented in Table 3. For the LDL outcome, older age (OR 0.94, per 10 years; 95% CI 0.91–0.97; p < 0.01) was associated with worse guideline concordance. In contrast, diabetes (OR 1.44; 95% CI 1.35–1.54; p < 0.01), total number of prescribed medications (OR 1.01, per 1 medication; 95% 1.00–1.01; p < 0.01), total number of outpatient visits (OR 1.02, per 1 visit; 95% CI 1.01–1.02; p < 0.01), and recent cardiac hospitalization (OR 1.28; 95% CI 1.11–1.49; p < 0.01) were associated with better guideline concordance.

**Table 3:** Multivariable analyses for the association between the hypothesis variables and guideline concordance^*#^

**Hypothesis variables**	**LDL concordance (95% CI; p-value)**	**BP concordance (95% CI; p-value)**
**Patient factors**		
Age (per 10 year increment)	0.94 (0.91–0.97; p < 0.01)	0.86 (0.83–0.89; p < 0.01)
Distance to medical center (per 25 mile increment)	0.99 (0.99–1.00; p = 0.19)	0.99 (0.99–1.00; p = 0.30)
Diabetes	1.44 (1.35–1.54; p < 0.01)	1.01 (0.94–1.08; p = 0.89)
Vascular disease^&^	0.99 (0.91–1.07; p = 0.76)	0.91 (0.84–0.99; p = 0.03)
**Processes of care**		
Total medications (per 1 medication increment)	1.01 (1.00–1.01; p < 0.01)	1.01 (1.00–1.01; p = 0.03)
Total Visits (per 1 visit increment)	1.02 (1.01–1.02; p < 0.01)	1.02 (1.01–1.02; p < 0.01)
Recent cardiac hospitalization	1.28 (1.11–1.49; p < 0.01)	1.34 (1.14–1.57; p < 0.01)
**Structures of care**		
Clinic volume	1.01 (1.00–1.02; p = 0.76)	0.82 (0.68–0.99; p < 0.01)
On-site cardiologist	1.14 (1.00–1.30; p = 0.04)	1.10 (0.99–1.22; p = 0.10)

*The cumulative logit model estimates the probability that each subject is in a lower (i.e., better) category compared to a higher (i.e., worse) category of guideline concordance. The reported odds ratio is the probability of being in a lower category (i.e., better concordance) compared to a higher category (i.e., worse concordance). The outcome categories for LDL were the following: 0) LDL measurement and LDL <100 mg/dl, 1) LDL measurement, but LDL≥100 mg/dl, and 2) no LDL measurement. The outcome categories for BP were the following: 0) SBP<140 and DBP<90 mm Hg; 1) SBP≥140 or DBP≥90 mm Hg, and 2) no BP measurement.

#Candidate covariates included all Table 1 and 2 variables.

&Presence of cerebrovascular and/or peripheral vascular disease

For the BP outcome, older age (OR 0.86, per 10 years 95% CI 0.83–0.89; p < 0.01) and the presence of vascular disease, cerebrovascular and/or peripheral vascular disease (OR 0.91; 95% CI 0.84–0.99; p = 0.03) were both associated with worse BP control. In contrast, total number of medications (OR 1.01, per 1 medication; 95% CI 1.00–1.01; p = 0.03), total number of outpatient visits (OR 1.02, per 1 visit; 95% CI 1.01–1.02; p < 0.01), and recent cardiac hospitalization (OR 1.34; 95% CI 1.14–1.57; p < 0.01) were associated with better BP concordance.

For the structures of care analysis, there was a trend for an association between on-site cardiology and better LDL (OR 1.14; 95% CI 1.00–1.30; p = 0.04) and BP (OR 1.10; 95% CI 0.99–1.12; p = 0.10) guideline concordance. In contrast, higher clinic volume was associated with worse BP concordance (OR 0.82; 95% CI 0.68–0.99; p < 0.01).

**Figure 2:**
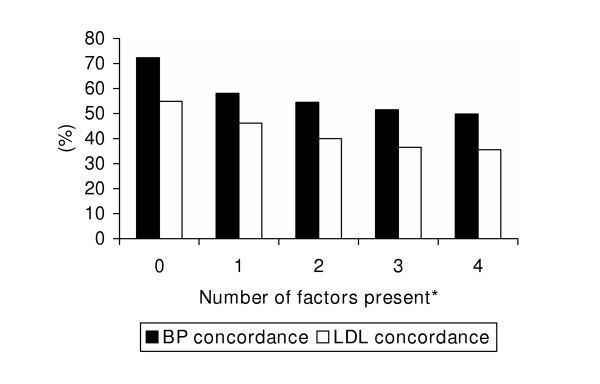
Guideline concordance based on the number of factors present. Factors: age≥65, presence of vascular disease, lack of diabetes, lack of recent cardiac hospitalization. p < 0.01 for trend for BP and LDL cholesterol concordance.

Finally, there was a graded inverse relationship between the number of factors present (i.e., age≥65, presence of vascular disease, lack of diabetes, and lack of recent cardiac hospitalization) and guideline concordance. LDL concordance ranged from 55% for those without any of the identified factors to 36% for those with all 4 factors (Figure 2). Similarly, BP concordance ranged from 72% for those without any factors to 50% for those with all 4 factors. Achievement of both LDL cholesterol and BP goals (data not shown) ranged from 47% when no factors were present to 31% when all 4 factors were present (p < 0.01 for trend for all).

## Discussion

The objectives of this study were to assess guideline concordance for secondary prevention care based on LDL cholesterol and blood pressure control and to identify specific patient, processes of care and structures of care factors associated with guideline concordance. We found that achievement of guideline recommendations for LDL (<100 mg/dl), and BP (<140/90 mm Hg) were 38.9%, and 53.4%, respectively. When concordance was assessed more globally, based on both LDL and BP control, less than one-quarter of patients were at target.

Several factors were associated with guideline concordance based on our multivariable analyses. Patient factors including older age and vascular disease were associated with worse guideline concordance, while diabetes was associated with better guideline concordance. Processes of care measures, including higher total number of medications, higher total number of outpatient visits, and recent cardiac hospitalization were associated with better guideline concordance. Finally, clinic volume and on-site cardiology were associated with guideline concordance; however, given the small number of facilities (n = 8), and the fact that many of the structural characteristics were highly correlated with each other, definitive conclusions cannot be made regarding the structures of care variables.

In contrast to prior studies, we focused exclusively on patients with known IHD and assessed guideline concordance for both hyperlipidemia and hypertension. Prior studies evaluating concordance with guideline recommendations for secondary prevention measures have mainly focused on either LDL cholesterol or blood pressure in any one study and not both of these co-morbidities together. In previous studies, rates of achieving LDL <100 mg/dl among those with a LDL measurement have ranged from 30–50% [2,3,16] with significant variability depending on the patient population and study setting. For hypertension control, studies have not exclusively focused on patients with known coronary disease, who comprised only up to 30% of the patients in any one of the studies. In these studies, blood pressure was controlled in only approximately 40% [4,5,17] of the population. Pedrinelli, et al. recently evaluated global risk in a cohort of hypertensive patients and noted low rates of achievement of both LDL cholesterol and BP targets. Among the highest risk patients, those defined as having a 10 year risk for a IHD event >20% or those with known IHD, BP averaged 170/110 mm Hg and the mean LDL cholesterol was 148 mg/dl [18]. Together, these studies highlight a need to apply a global approach to risk factor modification, especially among those with known IHD, who are at high risk for recurrent cardiovascular events.

Rates of guideline concordance for LDL cholesterol and BP noted in the current study are similar to, if not better than those reported in the literature, and are consistent with prior studies supporting the quality of VA care compared to non-VA settings. For example, Jha, et al. compared VA to Medicare fee-for-service for multiple outpatient chronic conditions and found that the VA outperformed Medicare fee-for-service on 12 of 13 indicators in the year 2000 [19]. For lipid screening among diabetics, the rate was significantly higher among veterans compared to the Medicare fee-for-service patients (89% vs. 60%). Asch, et al. also noted better performance on many of the quality indicators for outpatient care among VA patients compared to a national community sample [20]. The adjusted rates of adherence to hyperlipidemia and hypertension indicators were 64% and 78%, respectively for VA patients compared to 53% and 65%, respectively for the community sample patients. These studies, along with the findings of the current study, thereby support a higher level of quality of care among veterans in the outpatient setting, although significant room for improvement remains.

Overall, achievement of secondary prevention measures, both within and outside of the VA, remains suboptimal. Among patients above LDL goal, 38% of the patients were not prescribed any lipid-lowering medications and among patients above BP targets, 9% of the patients were not prescribed any antihypertensive medications in our study. Conditions such as hyperlipidemia and hypertension are often asymptomatic and less likely to be emphasized during outpatient visits that are frequently centered on acute problems and conditions. In addition to intensifying medical therapy (i.e., starting new medications or increasing dosages of existing medications), current processes of care can be modified to improve control of these measures. For example, computerized reminders directed at care providers [21,22] as a component of a multi-faceted intervention can be implemented to identify high-risk patients (e.g., high-risk based on the factors identified in this study) requiring aggressive treatment. In addition, outpatient visits, following the Chronic Care Model paradigm [23], can be planned to specifically focus on secondary prevention measures. These planned visits would allow care providers the necessary time to titrate therapy for these conditions. Similarly, disease management programs targeting secondary prevention measures can be established to complement usual care. Initial studies of disease management programs for various cardiovascular conditions [24] have been promising. Prospective rigorously designed studies are needed to determine whether changes to current care processes can improve LDL cholesterol and BP control among IHD patients.

Furthermore, interventions directly targeting patients can be implemented to improve self-management and increase patient adherence to recommended therapies. Telehealth technologies, such as interactive voice response, the Internet or Health Buddies [25,26] (i.e., an in-home technology device) can be incorporated into routine patient follow-up. These technologies can increase patient contact with the healthcare system, without adding to clinic visits. Interactive voice response (IVR) technology has been shown to increase patient adherence with medications, as well as improve some intermediate outcomes such as blood pressure and glycemic control [25]. These telehealth technologies should be examined prospectively in clinical trials to determine if the addition of these technologies to usual care improves guideline concordance.

There are several potential limitations to this study. This was a 15-month cross sectional study and we cannot rule out the chance that the outcome (poor LDL or BP control) affected processes of care due to measurement overlap. However, our main objective was to identify an association between these factors and not to establish temporal associations between the independent and dependent variables. Second, we arbitrarily defined a timeframe to assess for the presence of outcomes. Patients could have had a measurement just prior to or after the defined time periods. The defined time periods however, were longer than guideline recommendations for follow-up of LDL cholesterol and BP [6,14] even among patients who have achieved target goals. Third, although the database included complete laboratory, pharmacy records, and visit histories from all Pacific Northwest VA facilities, information from outside the VA system was not available. Nevertheless, patients in the study had an average of 9 clinic visits during the study period and were prescribed an average of 11 medications and it was likely that we captured a significant proportion of all health care utilization by these patients. Lastly, the guideline targets used for the study were applicable to both patients with and without known IHD. The BP targets were SBP<140 and DBP<90 mm Hg [14], which are the same for patients without known IHD. Therefore, the blood pressure goals established for this study should be achieved in all patients regardless of ischemic heart disease status.

## Conclusion

We found that less than one-quarter of patients with known ischemic heart disease have achieved both LDL cholesterol and BP targets as recommended by national guidelines. Patients at the highest risk for guideline non-concordance can be identified based on four factors, including age≥65, presence of vascular disease, lack of diabetes and lack of recent cardiac hospitalization. Quality improvement efforts are urgently needed to improve upon these secondary prevention measures to reduce the morbidity and mortality of patients with known ischemic heart disease.

## List of abbreviations

ACS: Acute Coronary Syndrome

BP: Blood pressure

CABG: Coronary Artery Bypass Graft

DBP: Diastolic blood pressure

IHD: Ischemic heart disease

ICD-9: International Classification of Diseases-9th revision diagnosis

LDL: Low density lipoprotein

OPC: Outpatient Care File

PTF: Patient Treatment File

PCI: Percutaneous Coronary Intervention

SBP: Systolic blood pressure

VA: Department of Veteran Affairs

## Competing interests

The author(s) declare that they have no competing interests.

## Authors' contributions

All authors contributed to the conception, design, and interpretation of the data. PMH performed the statistical analysis and drafted the article. All authors were involved in the revision. All authors gave final approval of the version to be published.

## Pre-publication history

The pre-publication history for this paper can be accessed here:


